# Folie à Deux in the Setting of COVID-19 Quarantine

**DOI:** 10.1155/2022/2046436

**Published:** 2022-04-20

**Authors:** Maxsaya Baez Nuñez, Ezequiel Rodriguez, Matthew DeLuca, Krishan Chirimunj, Monica Dhingra

**Affiliations:** ^1^St. George's University School of Medicine, Grenada WI; ^2^Bergen Newbridge Medical Center, Department of Psychiatry, Paramus, NJ, USA

## Abstract

We relay the case of a middle-aged male and his mother, an elderly female, who presented with *folie à deux* in the context of shared delusions of persecution and somatization during the COVID-19 quarantine period. The delusions were described as electric microwave shocks being transmitted to their internal organs by neighbors, followed by somatic symptoms of palpitations, headaches, and a shock-like perception. To the best of our knowledge, there have not been any reports that describe the development of *folie à deux* in the setting of the COVID-19 quarantine. *Folie à deux* may be defined as delusions affecting two or more individuals, usually first-degree relatives. Delusions classically transmit from one person, coined *the inducer*, to one or several individuals, *the induced*, who share and may expand on the communicated delusions. The preconditions that must exist for *folie à deux* to develop are an intimate emotional association between the inducer and the induced and a genetic predisposition to psychosis, such as blood relations with primary relatives. Isolation from society has also been considered a potential risk factor for shared psychosis in the recent literature. Given that to the best of our knowledge, there have not been any reports describing the development of *folie à deux* in the setting of the COVID-19 quarantine, the authors aim to dissect how extended periods of shared isolation from society during such a significant time in history may have served as a significant precipitating factor in the onset of shared psychotic disorder, while simultaneously illustrating a parallel relation to how such conditions may predispose certain subgroups to similarly dynamic-based mental health disorders. In addition, an evaluation of the origins and multifactorial etiology of *folie à deux*, along with that of existing treatment modalities, and the emphasis on advancement toward more effective treatment approaches will be provided.

## 1. Introduction

Shared psychotic disorder, commonly referred to as *folie à deux*, was first discussed in 1860 by Baillarger, followed by Lasègue and Falret in 1877. It was described as the appearance of concurrent psychotic symptoms in members of a family while living together and its transmission from an affected person, coined the *inducer*, to one or several unaffected individuals, the *induced*, who may elaborate on the communicated delusions [1, 2]. Since its introduction, there has been almost no change in the description of the phenomenon itself. Nonetheless, as its biologic and psychodynamic bases are limited to nonquantifiable methods—leaving room for hypothesis and intrigue—it has historically been an extensively discussed topic. Further understanding of its psychopathological mechanism may shed light to aspects underlying an array of unhealthy symbiotic interpersonal relationships. A consistency noted throughout the literature is that an intimate association between the inducer and induced serves as the unconditional basis to *folie à deux* [3]. A genetic predisposition to psychosis has been hypothesized to play an important role in the development of delusions among the induced. Further investigation into the literature also leads us to believe that one of the major risk factors for *folie à deux* is segregation from society [4]. The social distancing and quarantine experienced throughout the COVID-19 pandemic may serve to illustrate how isolation can affect mental health at a larger scale. During the mandated quarantine, we saw a significant increase in mental-health related ED visits [5, 6]. The pandemic may have had effects on people with, or at risk of psychosis. Furthermore, isolation may have reinforced unhealthy relationships during times of extreme closeness within one household, creating the optimal predisposing environment for the onset of shared psychotic disorder.

## 2. Case Presentation

Our primary patient—the *inducer*—was a middle-aged male who was brought into the emergency department by local police. He was endorsing persecutory delusions that his neighbors were transmitting electrical microwave shocks to his organs, specifically his brain and heart which lead to somatic symptoms of palpitations, headaches, and a shock-like sensation. Until the beginning of March, he recalls being in his usual state of health. He was diagnosed with COVID-19 prior to the quarantine mandate but had fully recovered. It was not until the social distancing and quarantine rules were set in place where he began experiencing new onset persecutory delusions. At the time, he had been living with his parents whom he quarantined with throughout the duration of the pandemic. The patient endorsed feeling heightened stress and anxiety about the COVID-19 virus. Although not reported, this patient had an extensive unclear psychiatric history. However, as per patient endorsement, as well as that of his collateral (father), this episode was singular in nature, as no such symptoms were ever exhibited by the patient in the past. This patient presentation is classified as delusional disorder, persecutory type, according to DSM-5.

Our secondary patient—the *induced*—was our primary patient's mother, an elderly woman who also began to endorse identical persecutory delusions approximately one week following her son's onset. The patient's mother had been isolated with the patient (her son) and husband primarily to avoid infection and because of the social distancing mandate. After several weeks of experiencing the delusions, she made an attempt to report her neighbors but instead alerted the police to their behavior and she was concurrently brought into the emergency department with her son. After careful police investigation, no evidence was present to sustain the accusations made and they deemed medical evaluation was warranted. She firmly stated, “My neighbors are sending electrical waves to our body.” Conversely, the father in the family did not share such ideations and served as a collateral for their individual assessments. This patient's records, own endorsement, and collateral also supported this episode being singular and may also be classified as a delusional disorder, persecutory type. Since the onset of their delusions, the mother and son have been able to take care of their activities of daily living, hygiene, and appetite without any reported changes. However, they have displayed various compensatory behaviors via sleeping outside their bedrooms with tin foil coverings in order to protect themselves from the transmitted electrical microwaves.

Throughout the interview process, the patient was calm and cooperative, although he did endorse feeling worried due to the nature of his current situation. When asked about prior visits to the emergency department, the patient did not recall full details. Upon medical chart review, a history of outpatient psychiatry visits was detailed. When asked about these visits, the patient did not remember the reason for them and was unaware if he was ever prescribed any psychiatric medications. Of note, approximately 30 years prior, he lost his job due to a work incident requiring psychiatric evaluation. He was not hospitalized at that time and reports never being hospitalized. The patient is a high school graduate, has been unemployed most of his life, and spends most of his time at home. He does not have any significant psychiatric family history and a past medical history of Tetralogy of Fallot, for which he received surgical intervention at childhood.

The patient's mother was also calm and cooperative during her visit but endorsed feeling scared due to the contextual situation of being in the emergency room and for leaving her husband at home without an aide. Based on the psychiatric evaluation, neither patient appeared to be in imminent danger to self, others, or property and did not meet criteria for inpatient hospitalization. After extensive discussion with the collateral, he felt comfortable accepting the patients' reintegration to their home life without any concern for safety, under the recommendation that they would benefit from outpatient psychiatry and therapy. Of note, patient identifiable data was excluded from this case report; therefore, no written consent was obtained from the patients.

## 3. Discussion

An important point to consider when evaluating the inciting factors of psychosis is the unique susceptibility of each individual patient to develop psychiatric symptoms. Cases of shared psychotic disorder, also known as *folie à deux*, are particularly rare in clinical practice. Special attention should be paid to determining the factors which led to the onset of the shared psychosis. As such, the clinician should also attempt to identify the relationship between the *inducer* and the *induced*. According to Lazurus, two preconditions must exist before *folie à deux* can develop: an intimate emotional association between the *inducer* and the affected person and a genetic predisposition to psychosis, such as blood relations with the primary patient [7]. Although debated, social isolation has also been considered a potential risk factor for shared psychosis in recent literature [2, 8]. As of 2020, the existing literature on clinical, diagnostic, and therapeutic aspects of folie a deux describes 7 reported cases documented in the timeframe between 1995 and 2019, wherein social isolation was mentioned as a major risk factor, with a prevalence higher than 60% in the listed cases (Arnone et al., 2006; Silveira & Seeman, 1995) [9].

To the best of our knowledge, there have not been any previous reports which describe the development of *folie à deux* in the setting of the isolation during the COVID-19 quarantine. With the discussion of this case, the authors aim to evaluate the contributions of the isolation during the COVID-19 pandemic on the development of shared psychosis, while illustrating a parallel relation to how such conditions may predispose certain subgroups to similarly dynamic-based mental health disorders. The authors will also provide a thorough evaluation of the origins and multifactorial etiology and previously documented treatment approaches of *folie à deux*.

Prior to the use of the term “shared psychotic disorder,” the nomenclature of this disease was based on the number of individuals that shared the same delusion. Among two, three, and even ten people, it was called *folie à deux*, *folie à trios*, and *folie à dix*, respectively. In rare instances, an entire family can share the same delusion, which was referred to as *folie à famill*e. Medical literature has described the tendency for those affected by *folie à deux* to be family members in a significant proportion of reports—as was evident in our case. As such, the idea that a genetic predisposition exists has been frequently discussed in the literature [2–4, 8]. Although *nature* cannot be fully excluded as a contributor, there should still be an emphasis placed on the ways in which *nurture* may help precipitate this disease. For example, the extent and duration of social proximity, as well as the intimacy of the relationship between affected individuals, must be taken into account. To that end, several cases of the shared psychotic disorder have been described among interpersonal relationships that were both spousal and interprofessional in nature [3, 10]. Importantly though, there are also extensive psychological factors that must be accounted for in this case.

Psychologically, the COVID-19 pandemic had a profoundly detrimental effect on the mental health of many patients. What is more, some patients were left without access to mental health resources during a time with increased psychological stress. Individuals and families were also isolated from society. The lack of external input likely hampered the ability for a person to conduct reality monitoring as it became difficult to determine the difference between internally self-generated information and externally derived information [11]. These precipitating factors may have the potential to contribute to the development of a psychotic episode. It is true that an intimate relationship, especially between immediate family members, has been cited as a contributing factor that increases the risk of developing shared psychotic disorder [2]. However, further research is needed to evaluate how the grouped isolation experienced during the COVID-19 pandemic correlates with the onset of shared psychosis. Additionally, it would be of value to know the prevalence of cases such as ours. Understanding this can give us insight into identifying potential cases of shared psychotic disorder and other mental health impacts that can occur because of isolation. A major hindrance to the study of this particular topic is the innate ethical and morale issues that are introduced when researchers propose removing physical and social stimuli from human beings.

A final consideration, in this case, is the evaluation of the *inducer* and the *induced.* Although not reported, our primary patient—the *inducer*—had an extensive psychiatric history. He also endorsed struggling to maintain an average socioeconomic status throughout the course of his life. Interestingly, after his SARS-CoV-2 infection resolved, he began to endorse new-onset persecutory delusions. There is little data to suggest a correlation between SARS-CoV-2 and detrimental neurotoxic effects, but numerous studies have shown the deleterious mental health effects of the pandemic [6, 12–16]. In our case, the innate psychosocial characteristics of the inducer, coupled with the circumstances surrounding the COVID-19 pandemic, likely contributed to the onset of psychosis in the inducer.

Our secondary patient—the *induced*—was an elderly mother who did not report any previous psychiatric history. It should be noted that we also considered her advanced age as a potential organic contributor to psychiatric disease. There is extensive literature available detailing age-related cognitive decline. Murman and Daniel explains, “Concept formation, abstraction, and mental flexibility decline with age, especially in subjects older than age 70” [17]. Along with this decline in cognition, we see a reciprocal increase in mental health-related issues. This coupled with the effects of the COVID-19 quarantine could have been the driving forces in our patient becoming *induced*. Kumar et al. mention age as an organic factor in the development of delusions among the *induced*, but greater etiological importance is still placed on the psychological and environmental factors involved in the presentation [2]. Given the patient's postmenopausal state, it is also appropriate to consider the potential contribution of decreased estrogen levels as a factor. Estrogen has been proposed as protective for salience in neuronal circuits, alongside that of brain aromatase, for which recent research has shown a regulatory role in synaptic activity, plasticity, neurogenesis, and the response of neural tissue to injury, mood, and cognition [18, 19].

Treatment modalities for approaching shared psychosis have also been controversial in the literature, as in most cases only one of the two affected individuals presents for clinical assessment and treatment. While the approach of separating both affected individuals has been suggested to be not only unrelated to clinical remission but also have an increased risk of adverse outcomes, most literature agrees that this separation approach is a crucial intervention in the management of the shared delusional disorder [19]. Regarding pharmacological approaches, the consensus is that antipsychotics have held positive outcomes; however, antidepressants have also been reported to yield desirable outcomes in some cases (Arnone et al., 2006; Kraya & Patrick, 1997). In most cases, inpatient treatment followed by the transition to outpatient community treatment of both, the inducer and induced, yielded the most desirable outcomes [20].

Further research leading to a better understanding of pathogenesis could lead to more effective treatment approaches. Because of the complex nature of the disease, it is very difficult to determine the exact mechanism by which a shared psychotic delusion can occur. Our case presents the idea that along with close proximity and intimacy being some of the most important preconditions, social isolation, such as the one experienced during the COVID-19 quarantine, may be a powerful catalyst for the development of shared psychotic disorder. The authors aim to highlight the need for further research that should investigate the role of social isolation in the development and expression of such disease. Strong emphasis should also be placed on understanding the nature of the relationship between all those in close proximity of those affected but who were spared of the delusion such as in the case of our presented patient's father, who, despite being in isolated quarantine with the two affected individuals reported, did not share the aforementioned delusions.

In conclusion, when evaluating the possible precipitating factors of a case of *folie à deux*, the multifactorial etiology quickly becomes evident. The perfect storm conditions necessary to create such an event are extremely rare. A recurrent theme is present among our presentation and other published cases. The shared delusion is between two immediately related individuals who are also in close proximity. The relationship between the *inducer* and the *induced* is very intimate. They are isolated from society, albeit not out of their own volition. In the recent literature, this is believed to be one of the greatest factors in the development of the shared psychotic disorder. Suresh et al. emphasize “This had cut off all his connections with reality and catalyzed the development of the paranoid delusions” [2]. In contrast, there are features of our case that make it unique. To our knowledge, there have been no previously reported cases of shared psychotic disorder developed during the COVID-19 pandemic. Despite reports of the development of individual psychosis post-COVID infection or during the quarantine isolation period, our case was centered around the individuals sharing identical psychoses under the psychological stressors of being infected with a potentially deadly virus and the extensive time isolated from society in lieu of the pandemic. In addition, the psychiatric history of our primary patient along with the fact the *inducer* and *induced* are relatives who live and are quarantined together could lead us to believe there is a natural aspect involved. Yet it is worth noting that the primary patient also lived with his father, the *induced* patient's husband, who never endorsed the shared delusions. This fact could mean the *induced* is genetically more susceptible to psychosis, but a review of literature places greater importance on the relationship dynamic rather than genetics [2–4].

## Data Availability

All supporting literatures were cited, respectively. Feel free to contact Maxsaya Baez Nuñez, MD, at mbaeznunez@newbridgehealth.org, if any queries should arise.
